# Fully Quantum Modeling of Exciton Diffusion in Mesoscale Light Harvesting Systems

**DOI:** 10.3390/ma14123291

**Published:** 2021-06-14

**Authors:** Fulu Zheng, Lipeng Chen, Jianbo Gao, Yang Zhao

**Affiliations:** 1 Bremen Center for Computational Materials Science, University of Bremen, 28359 Bremen, Germany; fzheng@uni-bremen.de; 2 Max Planck Institute for the Physics of Complex Systems, Nöthnitzer Str., 38, 01187 Dresden, Germany; lchen@pks.mpg.de; 3 Center for Geodata and Analysis, Faculty of Geographical Science, Beijing Normal University, Beijing 100875, China; jbgao.pmb@gmail.com; 4 Institute of Automation, Chinese Academy of Sciences, Beijing 100190, China; 5 School of Materials Science and Engineering, Nanyang Technological University, Singapore 639798, Singapore

**Keywords:** excitation energy transfer, superdiffusion, light harvesting complexes, fractal analyses, graphic processing units

## Abstract

It has long been a challenge to accurately and efficiently simulate exciton–phonon dynamics in mesoscale photosynthetic systems with a fully quantum mechanical treatment due to extensive computational resources required. In this work, we tackle this seemingly intractable problem by combining the Dirac–Frenkel time-dependent variational method with Davydov trial states and implementing the algorithm in graphic processing units. The phonons are treated on the same footing as the exciton. Tested with toy models, which are nanoarrays of the B850 pigments from the light harvesting 2 complexes of purple bacteria, the methodology is adopted to describe exciton diffusion in huge systems containing more than 1600 molecules. The superradiance enhancement factor extracted from the simulations indicates an exciton delocalization over two to three pigments, in agreement with measurements of fluorescence quantum yield and lifetime in B850 systems. With fractal analysis of the exciton dynamics, it is found that exciton transfer in B850 nanoarrays exhibits a superdiffusion component for about 500 fs. Treating the B850 ring as an aggregate and modeling the inter-ring exciton transfer as incoherent hopping, we also apply the method of classical master equations to estimate exciton diffusion properties in one-dimensional (1D) and two-dimensional (2D) B850 nanoarrays using derived analytical expressions of time-dependent excitation probabilities. For both coherent and incoherent propagation, faster energy transfer is uncovered in 2D nanoarrays than 1D chains, owing to availability of more numerous propagating channels in the 2D arrangement.

## 1. Introduction

Photosynthesis is one of the most essential biological processes in which specialized light-harvesting complexes (LHCs) in green plants, sea algae and bacteria funnel photo-induced excitations to reaction centers (RCs) with nearly 100% efficiency for subsequent solar to chemical energy conversion [1,2]. Despite the huge variety of light harvesting organisms in nature, some basic excitation transfer principles are shared by the LHCs, and are subjects of extensive studies that are devoted to resolving underlying mechanisms of optimized energy transfer processes in natural photosynthesis [3,4].

In addition to native photosynthetic systems, many artificial supramolecular structures and nanodevices have been designed and synthesized with advanced fabrication techniques for light-harvesting purposes [5,6,7,8,9,10]. For instance, nanoarrays of light harvesting 2 (LH2) complexes have been engineered and long-range energy transfer was found in those systems. [9]. However, long-range energy transport in native light harvesting systems is rarely achieved as LH2 complexes are randomly positioned in chromatophore membranes while being surrounded by a complex protein environment [11,12]. Recently a 100-million-atom scale model of an entire chromatophore has been reported, followed by extensive molecular dynamics (MD) simulations and first-principle calculations [13]. An in-depth understanding of energy transfer in such mesoscale photosynthetic systems is of great importance for guiding the design of artificial light harvesting devices for sustainable energy production.

A great number of theoretical studies have been conducted on energy transport in various light harvesting systems. The classical Föster theory [14] assumes incoherent inter-pigment exciton hopping and neglects inter-chromophore coherence leading to probability master equations involving populations only. The Föster theory fails to explain recent two-dimensional electronic spectra (2DES) of photosynthetic systems, which were claimed to be direct evidence of quantum coherence through the observation of quantum beating signaling light-induced oscillations in both donors and acceptors [15,16,17]. A multichromophoric Förster resonance energy transfer theory was later proposed to describe exciton hopping between weakly coupled molecular groups, and applied to probe energy transfer in B800 → B850 [18] and B850 → B850 [19]. Coherent energy transport can be accounted for by the Redfield theory assuming weak electron-phonon coupling and a Markovian bath [20]. An accurate account of exciton dynamics in light harvesting systems goes beyond perturbative and Markovian approximations [1]. Much progress on non-perturbative and non-Markovian treatments has been achieved over past few years [21,22], such as the hierarchy equation of motion (HEOM) [23,24], the multilayer multiconfiguration time-dependent Hartree methods [22], and the hierarchy of pure states equations [25,26]. In order to circumvent numerical difficulties encountered by many nonperturbative approaches, the Dirac–Frenkel time-dependent variational principle has been adopted to investigate the real time exciton–phonon dynamics [27,28,29,30]. Compared to reduced density matrix approaches in which the degrees of freedom (DOFs) of the bath are traced out, wave function based methods can capture explicitly the bosonic DOFs, making it one of the most promising platforms to study combined exciton–phonon dynamics.

In contrast to experimental studies on photosynthetic systems with multiple LH2 complexes, most theoretical investigations have been carried out on a single LH2 complex [24,31,32,33,34]. Even for a slightly larger system, e.g., a pair of B850 rings [35], only few fully quantum mechanical simulations have been reported on the exciton dynamics [23,36,37]. Taking advantage of numerically efficient time-dependent variation with the Davydov trial states, Zhao and coworkers explored the inter-B850 exciton dynamics [36,37] and exciton diffusion in chlorosome with graphic processing units (GPUs) [38]. Excellent performance of our wave function based approach in exciton dynamics simulation of more than 300 pigments in chlorosome encourages the applicability of this method to larger systems, such as multiple LHCs in chromatophore membranes.

In this work, the exciton propagation in mesoscale photosynthetic systems, i.e., B850 nanoarrays, is investigated by the time-dependent variational method as well as the classical master equation. The remainder of the paper is organized as follows. In Section 2, we outline the theoretical framework of the Dirac–Frenkel time-dependent variational approach and the classical master equation method. The structures of the nanoarrays are also described in this section. Numerical results from the quantum coherent description and the classical master equation are discussed in Section 3. With efficient simulations performed on GPUs, exciton dynamics in B850 nanoarrays is obtained and a superdiffusive component in the short time region is found. The superradiance enhancement factor is recorded to quantify the exciton delocalization. Fractal analysis is performed on the exciton dynamics for the first time. Application of the classical master equation to the exciton diffusion in B850 nanoarrays is also demonstrated. Conclusions are presented in Section 4.

## 2. Model of Mesoscale LHCs and Methodologies

### 2.1. B850 Nanoarrays

**Figure 1:**
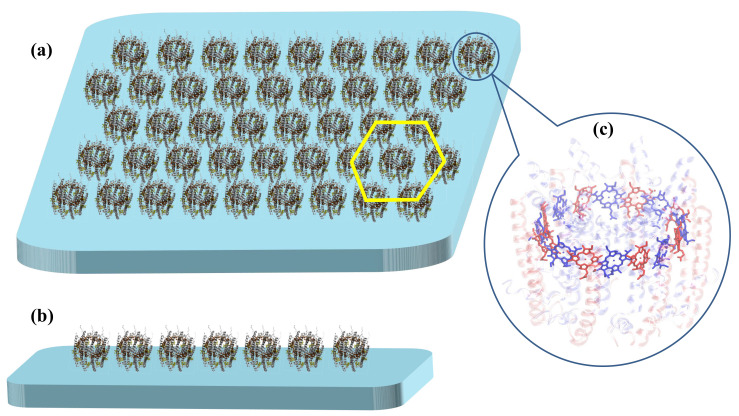
Schematic representation of the B850 nanoarrays used in this work to investigate the excitation energy migration: (**a**) two-dimensional (2D) and (**b**) one-dimensional (1D) B850 nanoarrays. The distance between adjacent B850 rings is 8 nm and each B850 ring is surrounded with six rings in the 2D nanoarray (as illustrated with the yellow hexagon in (**a**)). The arrangements of the bacteriochlorophyll *a* (BChl*a*) molecules in the B850 ring are illustrated in (**c**) with the proteins shown in transparent. The coordinates are extracted from the crystal structure of the LH2 complex (PDB ID: 1LGH) [41]. The α and β pigments are colored blue and red, respectively.

Taking LHCs in the chromatophore of purple bacteria as an example, multiple LH2 and LH1-RC complexes are randomly distributed on the membrane. A typical LH2 complex has a ring-shaped structure (see Figure 1), which consists of eight to ten subunits with each subunit containing two apoproteins (α− and β− proteins), three bacteriochlorophyll *a* (BChl*a*) molecules and one carotenoid [39,40,41]. Two of the BChls form a dimer and all dimeric BChls are densely packed to compose the B850 ring with the absorption band peaked at 850 nm. Coordinated with the apoproteins, two types of pigments in B850, i.e., α- and β-BChls, are colored blue and red in Figure 1c, respectively. As a consequence of large inter-pigment distances, the third BChls in the subunits mainly contribute to the absorption peak at 800 nm and form the B800 ring that is coaxial to (but displaced from) the B850 ring. Upon excitation, the excitonic energy is transferred from the B800 ring to the B850 ring within a single LH2 complex before migrating to nearby complexes [23,42,43]. Inter-LH2 energy transfer takes place between neighboring B850 rings. As one crucial step in the efficient energy transfer process in photosynthesis, the inter-complex energy propagation in purple bacteria is inadequately investigated, thereby deserving more attention.

As shown in Figure 1, two kinds of B850 nanoarrays, i.e., one-dimensional (1D) and two-dimensional (2D) nanoarrays are constructed to mimic the mesoscale LHCs and to study excitation energy transfer in this work. For 2D nanoarrays shown in Figure 1a, each ring is surrounded by six others. Considering the outer diameter of realistic LH2 complex is about 75 Å [41], the distance between two neighbouring B850 rings is set to 8.0 nm for both 1D and 2D nanoarrays.

### 2.2. Quantum Coherent Modeling for Exciton-Phonon Dynamics

The B850 pigments are densely packed, and strong inter-pigment excitonic interactions give rise to delocalized exciton over several chromophores [44]. The excitation energy transfer in B850 nanoarrays is modeled with the Holstein Hamiltonian [45,46]:

(1) $$\hat{H}={\hat{H}}_{\mathrm{ex}}+{\hat{H}}_{\mathrm{ph}}+{\hat{H}}_{\mathrm{ex}-\mathrm{ph}}.$$

Here

(2) $${\hat{H}}_{\mathrm{ex}}=\sum_{r_{1}r_{2}}\sum_{mn}J_{mn}^{r_{1}r_{2}}{\hat{a}}_{m}^{r_{1}†}{\hat{a}}_{n}^{r_{2}}$$

is the Frenkel-exciton Hamiltonian, and ${\hat{a}}_{n}^{r†}$ (${\hat{a}}_{n}^{r}$) is the exciton creation (annihilation) operator on the $n^{\mathrm{th}}$ site (with a total number of $N_{s}=16$ sites in one B850 ring) of the $r^{\mathrm{th}}$ ring (with a total number of $N_{r}$ B850 rings). $J_{mn}^{r_{1}r_{2}}$ is the excitonic coupling between pigment *m* on the $r_{1}^{\mathrm{th}}$ B850 ring and pigment *n* on the $r_{2}^{\mathrm{th}}$ B850 ring [47]. The diagonal elements in the exciton Hamiltonian *J* are the site energies of the B850 pigments and the computational details for *J* are described in Appendix A.

The exciton dynamics in B850 complexes is affected by environmental effects, such as conformational changes in pigments and surrounding proteins. In the Holstein Hamiltonian, the environment of the exciton is represented by a phonon bath, which is expressed as a set of harmonic oscillators

(3) $${\hat{H}}_{\mathrm{ph}}=\sum_{r}\sum_{q}\omega_{q}^{r}{\hat{b}}_{q}^{r†}{\hat{b}}_{q}^{r}.$$

where ${\hat{b}}_{q}^{r†}$ (${\hat{b}}_{q}^{r}$) is the creation (annihilation) operator for phonon with momentum *q* and frequency $\omega_{q}^{r}$ in the $r^{\mathrm{th}}$ ring. The exciton is coupled to the phonon bath via the exciton–phonon interaction Hamiltonian

(4) $${\hat{H}}_{\mathrm{ex}-\mathrm{ph}}=-\frac{1}{\sqrt{N_{s}}}\sum_{r}\sum_{n}\sum_{q}g_{q}^{r}\omega_{q}^{r}{\hat{a}}_{n}^{r†}{\hat{a}}_{n}^{r}\left(e^{iqn}{\hat{b}}_{q}^{r}+e^{-iqn}{\hat{b}}_{q}^{r†}\right).$$

Here $g_{q}^{r}$ is the exciton–phonon coupling strength and the Planck’s constant *ℏ* is set to unity.

A fully quantum mechanical description of both the exciton and the phonons can be afforded by the Davydov $D_{1}$ *Ansatz*

(5) $$|\Psi_{D_{1}}\left(t\right)\rangle =\sum_{r}\sum_{n}\alpha_{n}^{r}\left(t\right){\hat{a}}_{n}^{r†}{\hat{U}}_{n}^{r†}{\left(t\right)|0\rangle }_{\mathrm{ex}}{|0\rangle }_{\mathrm{ph}},$$

where

$${\hat{U}}_{n}^{r†}\left(t\right)=\exp\left\{\sum_{q}\left[\lambda_{nq}^{r}\left(t\right){\hat{b}}_{q}^{r†}-\lambda_{nq}^{r*}\left(t\right){\hat{b}}_{q}^{r}\right]\right\}$$

is the Glauber coherent operator and $\alpha_{n}^{r}\left(t\right)$ ($\lambda_{nq}^{r}\left(t\right)$) are the variational parameters representing exciton amplitudes (phonon displacements). The Dirac–Frenkel time-dependent variational approach is employed in this work to investigate excitation energy transfer in both 1D and 2D B850 nanoarrays from a fully quantum perspective. The Lagrangian formalism of the Dirac–Frenkel time-dependent variational principle is applied to obtain the equations of motion for the variational parameters [29,30,48,49], and detailed derivations are shown in Appendix B.

Many observables can be calculated from the time-dependent wave function obtained. The electronic population and coherence are included in the reduced single-exciton density matrix $\rho_{mn}^{r_{1}r_{2}}\left(t\right)$ which is obtained from a trace over the phonon bath

(6) $$\begin{array}{ccc} \rho_{mn}^{r_{1}r_{2}}\left(t\right) & = & {\mathrm{Tr}}_{b}\left[|\Psi_{D_{1}}\left(t\right)\rangle \langle \Psi_{D_{1}}\left(t\right)|{\hat{a}}_{m}^{r_{1}†}{\hat{a}}_{n}^{r_{2}}\right]\\ & = & \alpha_{m}^{r_{1}*}\left(t\right)\alpha_{n}^{r_{2}}\left(t\right)S_{mn}^{r_{1}r_{2}}\left(t\right), \end{array}$$

where the Debye-Waller factor $S_{mn}^{r_{1}r_{2}}\left(t\right)$ is

(7) $$\begin{array}{ccc} S_{mn}^{r_{1}r_{2}}\left(t\right) & = & \exp\left\{\sum_{q}\left[\lambda_{mq}^{r_{1}*}\left(t\right)\lambda_{nq}^{r_{2}}\left(t\right)\delta_{r_{1}r_{2}}-\frac{1}{2}|\lambda_{mq}^{r_{1}}{\left(t\right)|}^{2}-\frac{1}{2}{\left|\lambda_{nq}^{r_{2}}\left(t\right)\right|}^{2}\right]\right\}. \end{array}$$

The exciton population on the rth B850 ring can be obtained by tracing $\rho_{mn}^{r_{1}r_{2}}\left(t\right)$ over pigments comprising this complex

(8) $$\rho_{r}\left(t\right)=\sum_{m=1}^{N_{s}}\rho_{mm}^{rr}\left(t\right).$$

For exciton propagation, the mean square displacement (MSD) can be measured as

(9) $$\langle x^{2}\left(t\right)\rangle =\sum_{r=1}^{N_{r}}{\left(d_{r}\right)}^{2}\rho_{r}\left(t\right)$$

with $d_{r}$ being the distance between the rth B850 ring and the complex on which the exciton is initially localized. Usually, the MSD exhibits a power-law relation with time, i.e., $\langle x^{2}\left(t\right)\rangle \propto t^{\gamma }$. The exponent γ determines the characteristics of diffusion processes. If γ=1, the process is the Fickian diffusion. 0<γ<1 and γ>1 correspond to the subdiffusion and superdiffusion, respectively. In this work, the time evolution of the MSD is measured in order to characterize the exciton diffusion process in B850 nanoarrays.

To study the exciton delocalization in B850 nanoarrays, the coherence size $L_{\rho }$ is defined as [50,51,52]

(10) $$\begin{array}{c} L_{\rho }\left(t\right)={\left(\sum_{r_{1}r_{2}}\sum_{mn}\left|\rho_{mn}^{r_{1}r_{2}}\left(t\right)\right|\right)}^{2}{\left[N\sum_{r_{1}r_{2}}\sum_{mn}{\left|\rho_{mn}^{r_{1}r_{2}}\left(t\right)\right|}^{2}\right]}^{-1} \end{array}$$

where $N=N_{s}\times N_{r}$ is the total number of pigments in the system. The coherence size $L_{\rho }$ has been used as the exciton delocalization length in various photosynthetic systems, such as the LH2 [23,33,50,53,54,55,56,57] and the chlorosome [38]. $L_{\rho }$ quantifies the length scale over which $\rho_{mn}^{r_{1}r_{2}}$ decays along the anti-diagonal direction [50,55]. This quantity is related to the superradiance enhancement factor $L_{s}$ which is the ratio of radiative decay rate of a molecular aggregate to that of a monomer of the same type [50,51,55]. In a doorway-window representation, $L_{s}$ can be written as [50,55]

(11) $$L_{s}\left(t\right)=\sum_{r_{1}r_{2}}\sum_{mn}M_{mn}^{r_{1}r_{2}}\rho_{mn}^{r_{1}r_{2}}\left(t\right)$$

where $M_{mn}^{r_{1}r_{2}}=d_{m}^{r_{1}}\cdot d_{n}^{r_{2}}$ contains all the relevant geometric information of the system with $d_{m}^{r_{1}}$ being the unit vector of the transition dipole moment of pigment *m* on the $r_{1}^{\mathrm{th}}$ B850 ring.

### 2.3. Incoherent Inter-B850 Exciton Hopping

Ignoring energy transfer within a B850 ring and modeling the B850 complex as one single aggregate, one can apply the Förster theory to describe the excitation energy propagation in large B850 nanoarrays by treating inter-aggregate energy transfer as incoherent hopping of exciton. This model is also used to investigate excitation energy transfer in both 1D and 2D B850 nanoarrays in this work.

The exciton dynamics in 1D nanoarrays composed of B850 rings can be described with the classical master equation

(12) $$\begin{array}{c} \frac{dp_{i}}{dt}=r_{a}A_{i}-\left(K_{i}+\sum_{j}k_{ij}\right)p_{i}+\sum_{j}k_{ji}p_{j}, \end{array}$$

where the ring index *i* runs from 1 to $N_{r}$, $p_{i}$ is the unnormalized probability that ring *i* is excited, $K_{i}$ is the rate of local dissipation for ring *i*, $k_{ij}$ is the exciton transfer rate from ring *i* to *j*, $A_{i}$ is the profile of excitation laser, and $r_{a}$ is the rate of energy absorption by the B850 ring from the laser.

Assuming that the exciton transfer only occurs between two neighboring complexes with an inter-complex transfer rate *k* and that the dissipation rate is the same for all complexes $K=K_{i}$, the master equation Equation (12) is reduced to

(13) $$\begin{array}{c} \frac{dp_{i}}{dt}=r_{a}A_{i}-\left(K+2k\right)p_{i}+kp_{i-1}+kp_{i+1}. \end{array}$$

For the stationary case of $dp_{i}/dt=0$, Equation (13) has an analytical solution [58]

(14) $$\begin{array}{ccc} p_{i} & = & \frac{1}{k}\sum_{j=1}^{N_{r}}[\left(\cosh\left[(N_{r}+1-|j-i|)\lambda \right]-\cosh\left[(N_{r}+1-i-j)\lambda \right]\right)\\ & & \;\;\;\;\;\;\;\;\;\;/\left(2\sinh\lambda \sinh\left[(N_{r}+1)\lambda \right]\right)]r_{a}A_{j} \end{array}$$

where λ=arccosh[(K+2k)/2k]. In order to compare with the laser profile, the normalized excitation probability ${\tilde{p}}_{i}$ is calculated as

(15) $$\begin{array}{c} {\tilde{p}}_{i}=\frac{p_{i}}{p_{i_{0}}}. \end{array}$$

It is clear that ${\tilde{p}}_{i_{0}}=1$, and $r_{a}$ has no effects on ${\tilde{p}}_{i}$, which is set to be unity in the rest of this work. Equations (14) and (15) indicate that ${\tilde{p}}_{i}$ only depends on the ratio K/k and the excitation laser.

In a 2D B850 nanoarray, each complex is surrounded with six neighboring complexes. The master equation is written as

(16) $$\begin{array}{ccc} \frac{dp_{ij}}{dt} & = & r_{a}A_{ij}-\left(K+6k\right)p_{ij}+k(p_{i-1,j}+p_{i+1,j}\\ & & \;+p_{i,j-1}+p_{i,j+1}+p_{i-1,j+1}+p_{i+1,j-1}), \end{array}$$

where (*i*, *j*) is the B850 index in 2D nanoarray model. Stationary excitation probability distribution can be obtained by solving Equation (16) numerically with the constraint of $dp_{ij}/dt=0$.

In addition, analytical solutions can be obtained for exciton dynamics with laser produced initial conditions. For a B850 chain in the absence of a laser field ($A_{i}=0$), the excitation dynamics governed by Equation (13) has an analytical solution [59,60]

(17) $$\begin{array}{c} p_{i}\left(t\right)=e^{-(K+2k)t}\sum_{m=-\infty }^{\infty }p_{m}\left(0\right)I_{i-m}\left(2kt\right), \end{array}$$

where $I_{n}\left(x\right)=i^{-n}J_{n}\left(ix\right)$ is the modified Bessel function of the first kind. For the 2D B850 nanoarray, a similar solution can be derived from Equation (16) with $A_{ij}=0$,

(18) $$\begin{array}{ccc} p_{ij}\left(t\right) & = & e^{-(K+6k)t}\sum_{l=-\infty }^{\infty }\sum_{m=-\infty }^{\infty }\sum_{n=-\infty }^{\infty }p_{lm}\left(0\right)I_{i-n-l}\left(2kt\right)I_{j+n-m}\left(2kt\right)I_{n}\left(2kt\right). \end{array}$$

The initial condition $p_{m}\left(0\right)$ [$p_{lm}\left(0\right)$] for the 1D (2D) nanoarray can be prepared by turning on the laser field and then switching it off immediately. Then the excitation probability dynamics of a given complex can be obtained using Equation (17) [Equation (18)] for the 1D (2D) model.

## 3. Results and Discussion

### 3.1. Coherent Exciton Transfer in B850 Nanoarrays

The advent of ultrafast spectroscopy brought many breakthroughs in our understanding of energy transfer in natural photosynthesis. For example, the discovery of a long-lived beating in the 2DES of the FMO complex [15] triggered an avalanche of similar probes into excitation dynamics in a variety of photosynthetic systems, including LH2 complexes [42,61,62,63,64]. The oscillatory behavior has been attributed to quantum coherence, the origin of which is still under debate [5,44,65]. In the B850 ring of the LH2 complex, BChls are closely packed and the porphyrin rings of these molecules are nearly parallel [41], producing strong excitonic coupling between two adjacent pigments which may lead to exciton delocalization [33,44,66]. Therefore, a quantum description is essential to understand energy transfer in systems composed of B850 rings.

In the framework of the Dirac–Frenkel time-dependent variation, the Davydov $D_{1}$ *Ansatz* has been successfully applied to investigate zero-temperature exciton–phonon dynamics in the chlorosome [38] as well as in the single and double B850 complexes [36,37,67]. The extraordinary numerical efficiency and accuracy of this approach make it a promising methodology to study the exciton diffusion in mesoscale LHCs, such as B850 nanoarrays. In order to precisely describe the polaron dynamics in wider parameter regimes, a variational framework with the multiple Davydov states has been developed recently [68]. It has been reported that a large multiplicity (∼32) is needed for the multiple-$D_{2}$ trial state to reproduce comparable accuracies of a single Davydov $D_{1}$ *Ansatz* in the strong electronic coupling regime [68,69]. Parameterizing the initial phonon states with temperature-dependent phonon displacements, one can apply the time-dependent variational principle with the multiple Davydov states to describe system dynamics at finite temperatures for a broad range of various parameters, such as the system-bath coupling and the electronic coupling [69,70].

#### 3.1.1. Parameterization of the Phonon Bath

As shown in Hamiltonian (4), explicit values of $\omega_{q}^{r}$ and $g_{q}^{r}$ for individual bath modes are required in a proper description of the coupled exciton–phonon dynamics. Typically, these parameters are obtained by discretizing the bath spectral densities, which can be established from QM/MM calculations [53,71], or from fitting the fluorescence line-narrowing spectra [72,73] of the target system. It has been found that different methods can produce totally different spectral densities. For instance, in spectral densities calculated by MD simulations of some photosynthetic systems, several high frequency (∼1670 cm${}^{-1}$) modes are found to couple strongly to the electronic transitions [53,71]. On the other hand, the spectral density obtained by fitting the fluorescence line-narrowing spectra of the B777 complex includes some low frequency modes strongly coupled to the excitons [73]. With the same procedure, Novoderezhkin et al. constructed the LHCII spectral density with 48 effective modes in the frequency range from 100 cm${}^{-1}$ to 1700 cm${}^{-1}$ [72]. In addition to the spectral densities evaluated from simulations or experiments, some generalized schemes, such as the linear dispersed phonon modes [36,37,38,67] and the Drude spectral density [23], have been used to explain optical and transport properties in photosynthetic complexes.

**Figure 2:**
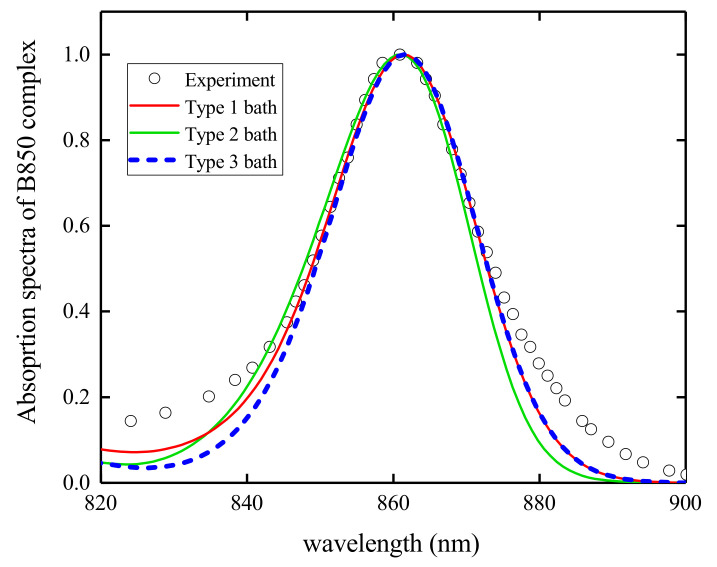
Calculated absorption spectra of the B850 complex with different phonon baths. Type 1 bath is characterized by 48 effective modes [72], type 2 bath is represented by a Drude spectral density, and type 3 bath is composed of a set of linear dispersed phonon modes. The experimental data are shown by open circles [74].

In order to study the effects of the bath spectral densities, three bath configurations are adopted here: (1) The 48 effective bath modes extracted from the LHCII spectral density [72] are used to model the first bath configuration; (2) The parameters for the second bath configuration are obtained by discretizing the Drude spectral density; (3) In the third case, a set of linear dispersed phonon modes are coupled to the excitons. A linear phonon dispersion $\omega_{q}^{r}=\omega_{0}\left[1+W(2|q|/\pi -1)\right]$ with a bandwidth W=0.1 and momentum $q=2\pi n_{q}/N_{s}\;\left(n_{q}=-7,-6,\cdots ,7,8\right)$ is adopted in this work. The exciton–phonon coupling strength is parameterized from the Huang-Rhys factor *S* with the relation $\frac{1}{N_{s}}\sum_{q}{\left(g_{q}^{r}\right)}^{2}\omega_{q}^{r}=S\omega_{0}$ where $\omega_{0}=1670$ cm${}^{-1}$ is the characteristic phonon frequency [53]. A fixed Huang-Rhys factor S=0.5 is used throughout this work. The absorption spectra of the B850 complex are calculated with above three bath configurations, and the results are compared with the experimental data in Figure 2. It can be seen that these phonon baths lead to similar absorption spectra, which are in agreement with the measured results. Different bath configurations may produce different exciton–phonon dynamics. The discussion of the effects from different phonon baths on the dynamics is beyond the scope of current study. In this work, the linear dispersed phonon modes are adopted in the dynamics simulations and the bath induced effects on exciton diffusion are presented in following sections.

#### 3.1.2. Computational Advantage of GPU

A fully quantum mechanical description of the exciton–phonon dynamics in a multiple-chromophore system is a computational challenge because a huge number of DOFs in the Hilbert space are involved in calculations. To tackle this problem, a numerically efficient CUDA algorithm is implemented here on the architecture of GPUs. As can be seen from the Davydov $D_{1}$ *Ansatz* in Equation (5), there are $N_{r}\times N_{s}$ and $N_{r}\times N_{s}\times N_{s}$ variational parameters for the exciton ($\alpha_{n}^{r}$) and phonon ($\lambda_{nq}^{r}$), respectively. In our implementations, these parameters are conveniently labeled by the indexes of the blocks and threads of the GPU cards. Specifically, the exciton amplitude parameters $\alpha_{n}^{r}$ are evaluated in a 1D grid with $N_{r}$ 1D blocks each containing $N_{s}$ threads, while a 1D grid with $N_{r}$ 2D blocks each containing ($N_{s}$, $N_{s}$) threads for the phonon displacement parameters $\lambda_{nq}^{r}$. In the calculations of the reduced density matrix $\rho_{mn}^{r_{1}r_{2}}$ and the Debye-Waller factor $S_{mn}^{r_{1}r_{2}}$, we adopt a 2D grid with ($N_{r}$, $N_{r}$) 2D blocks each containing ($N_{s}$, $N_{s}$) threads.

**Figure 3:**
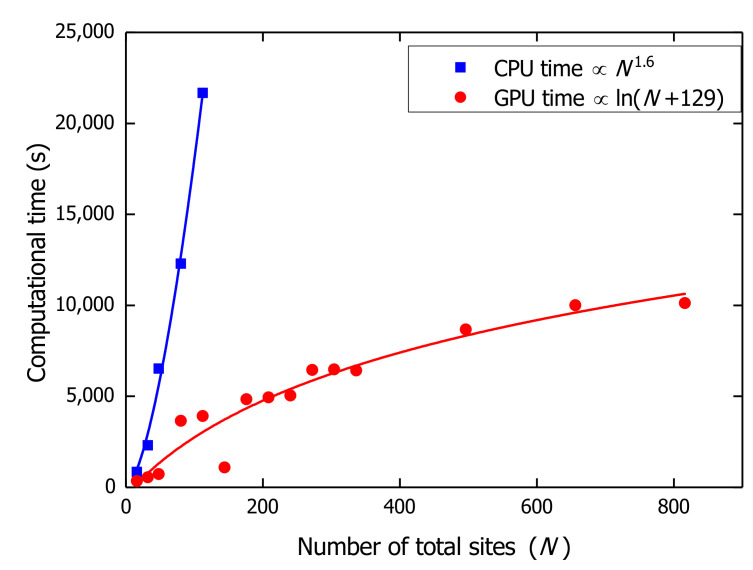
Computational time of CPU and GPU for different size of systems. The blue line is the power law fitting for the CPU time and the red line is the logarithmic fitting of the GPU time. The fitting parameters are shown in the legend with *N* being the total number of sites in the system.

To compare the computational efficiency between the CPU and GPU architecture, calculations for various B850 chains are done on CPU and GPU to obtain a 1 ps-long trajectory for each nanoarray. Computational times are compared in Figure 3 between CPU and GPU. The CPU computational time is found to increase with system size *N* obeying a power law ($\propto N^{1.6}$). The GPU architecture exhibits great advantages over CPU especially for larger system sizes, as the GPU computational time is a logarithmic function of system size [∝ln(N+129)]. With the efficient GPU algorithm, one can calculate the exciton dynamics in B850 chains composed of more than 100 B850 rings (more than 1600 sites). All quantum mechanical calculations in this work are carried out on the GPU platform.

#### 3.1.3. Effect of Initial Excitation Conditions

**Figure 4:**
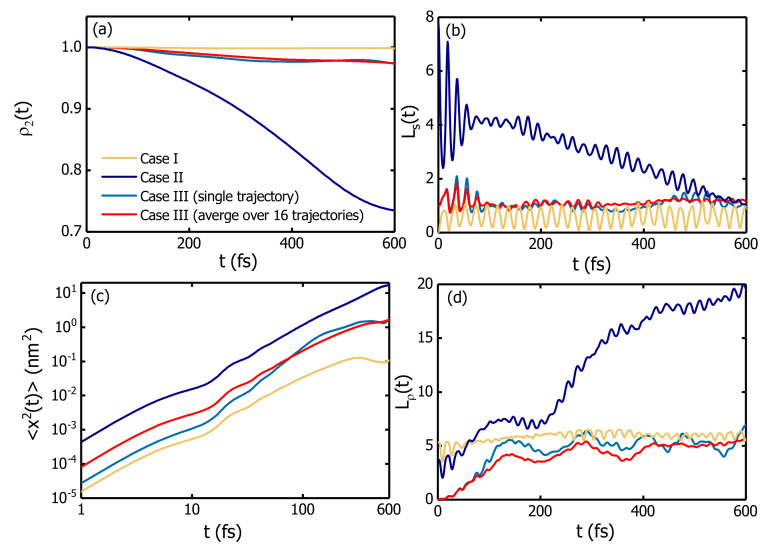
The exciton population on the central complex (**a**), the superradiance enhancement factor (**b**), the MSD (**c**), and the coherence size (**d**) for the exciton diffusion in a B850 chain with three B850 complexes. The calculations are performed with three initial excitation conditions, i.e., the lowest (Case I) and the second lowest (Case II) exciton states, and an individual pigment (Case III) of the central complex. For comparison, the results from a single trajectory (blue curves) of Case III are shown with the averaged results over 16 trajectories (red curves).

Exciton dynamics is first simulated in a three-ring B850 chain with the excitation initiated on the central B850. Three forms of excitation initiation are employed. In Cases I and II, the lowest and second lowest exciton state of the central ring are populated initially, respectively. In Case III, only one pigment on the central B850 is excited. For the third case, 16 trajectories are simulated and each trajectory is started from an individual pigment on the central complex. Results for three cases are shown in Figure 4. For Case III, averaged results from 16 trajectories are depicted in comparison with those from a single trajectory.

Shown in Figure 4 is the population dynamics of the central complex (a), the time evolution of the superradiance enhancement factor (b), the logarithmic plot of the MSD versus simulation time (c), and the temporal dependence of the coherence size (d). As illustrated in Figure 4a, the exciton is localized on the ring of initial excitation for a long time period if the central ring is initialized to be in its lowest exciton state (Case I), an optically dark state considering the transition dipole configurations of the B850 pigments. In contrast, the excitation energy transfer is most efficient in Case II where the exciton starts to propagate from the second lowest exciton state of the central complex. As the optically bright state which contributes to the absorption peak of the B850 ring, the second lowest exciton state is pairwise degenerate and exhibits the largest transition dipole moment among all the exciton states of the B850 aggregate, producing the most efficient excitation energy transfer. This finding is consistent with a previous report that an exciton state of the B850 ring with a larger transition dipole moment produces more efficient excitation energy transfer [75]. Comparing the exciton localization on the initial complex in Case I and the efficient energy transfer in Case II, one can safely draw the conclusion that the excitation energy is most likely transferred from the second lowest exciton state of a B850 ring to the neighboring complexes. Therefore, the second lowest exciton state of the central B850 ring is chosen as the initial conditions for the quantum mechanical calculations hereafter.

The log-log plots of the MSDs for three cases versus time are shown in Figure 4c, and linear relations with slopes ∼1.6 have been extracted indicating the superdiffusion behavior. In Case II, superdiffusion seems to last for about 600 fs in contrast to the time of 300 fs in Case I. Based on the averaged results from the 16 trajectories of Case III, the superdiffusive behavior of exciton holds for about 400 fs. This superdiffusive behavior is found for all the simulations irrespective of initial conditions, and is proposed to be an intrinsic property of exciton propagation in B850 nanoarrays.

#### 3.1.4. Exciton Delocalization

The superradiance enhancement factor $L_{s}$ and the coherence size $L_{\rho }$ are recorded during the simulations to characterize the exciton delocalization in B850 nanoarrays. As can be seen from its definition Equation (11), $L_{s}$ is dependent on the state and the transition dipole configuration of the system, quantifying the ratio of the radiative decay rate of the system to that of a single pigment [50,51]. In contrast, $L_{\rho }$ only contains the information of the reduced density matrix. As illustrated in Figure 4b, the superradiance enhancement factor $L_{s}$ in Case II has an initial value of 8. The large value of $L_{s}$ results from the initial state of this trajectory, i.e., the second lowest exciton state of the B850 aggregate which is one of the superradiant states of B850 [50]. For Case I in which exciton initiates propagation from the optically dark state ($L_{s}\left(0\right)=0$), the superradiance enhancement factor $L_{s}$ oscillates below 1.0 with the characteristic phonon frequency (20 fs) until the exciton starts to escape from the initial complex. As the oscillator strength of the initial dark state is almost zero, cooperative spontaneous emission of the whole system is nonexistent. For Case III where the exciton is initialized from one pigment, a superradiance enhancement factor oscillates around 1.0.

Compared to the superradiance enhancement factor $L_{s}$, the coherence size $L_{\rho }$ presents a different picture as shown in Figure 4d. At initial time, Case I has the largest coherence size because of nearly uniform excitation for the pigments in the central ring. With temporal evolution, $L_{\rho }$ oscillates around the value of 5 for Case I. Case II exhibits larger coherence sizes which cover more than 10 pigments. Special attention should be paid to the out-of-phase oscillations in the $L_{s}$ and the $L_{\rho }$ for Case II. The total transition dipole moment of the B850 complex is very small due to the nearly opposite orientations of the transition dipole moments of adjacent pigments. In this situation, the emission is induced by the loss of coherence. Therefore, the superradiance enhancement factor $L_{s}$ increases with the decrease of $L_{\rho }$ [50].

**Figure 5:**
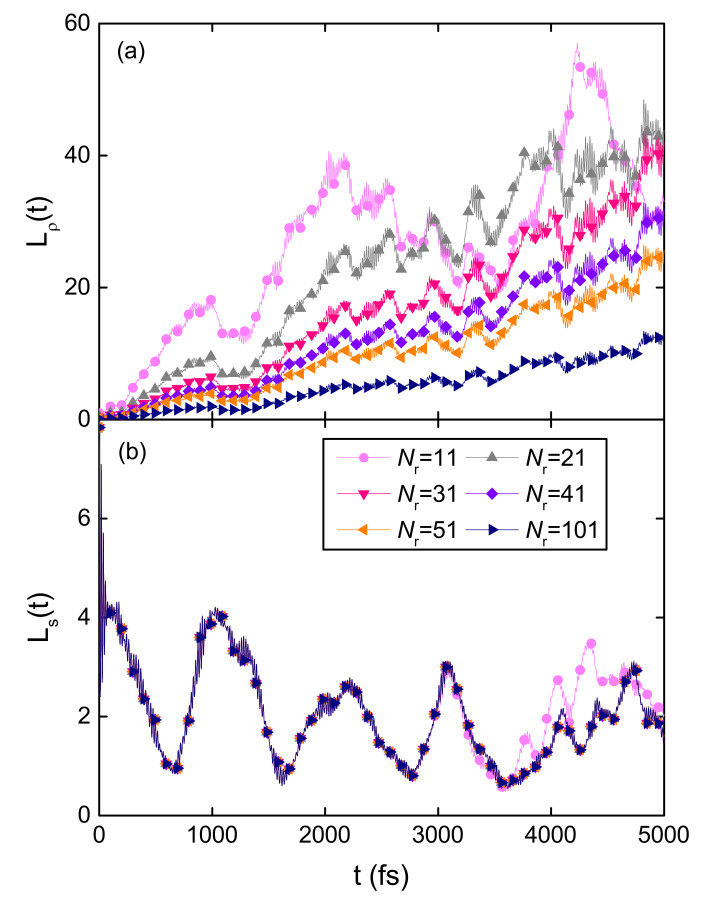
Time evolution of the coherence size $L_{\rho }$ (**a**) and the superradiance enhancement factor $L_{s}$ (**b**) for various B850 chains with the system sizes ranging from 11 to 101 rings. Initially the central ring of the B850 chain is excited to its second lowest exciton state.

To demonstrate the difference between $L_{s}$ and $L_{\rho }$, the exciton dynamics in several B850 chains with different system sizes is simulated and the initial excitation is localized on the central ring for all the simulations. The sizes of the chains vary from 11 to 101 B850 rings and the time evolution of the coherence sizes for these systems is shown in Figure 5a. Before the exciton arrives the boundary of the shortest chain ($N_{r}=11$), the exciton propagation in various chains is very similar, so the differences in $L_{\rho }$ are induced by the system size *N* in the denominator of Equation (10). As shown in Figure 5a, larger systems have smaller coherence sizes within 3 ps. At long times, the coherence sizes oscillate around an equilibrium value which is about half of the system size. In contrast to $L_{\rho }$, the superradiance enhancement factor $L_{s}$ is independent on the system size. The temporal evolution of $L_{s}$ for different B850 chains is depicted in Figure 5b. In the first 3 ps, all the systems exhibit the same behavior of $L_{s}$. Subsequently, the superradiance enhancement factor $L_{s}$ in the chain with 11 rings shows some visible deviations from those in other systems. It stems from the fact that the wave function spreads to the boundary of the 11-ring chain by 3 ps. The gradual decrease of $L_{s}$ is induced by the phonon induced decoherence. At long times, $L_{s}$ oscillates around 2 and agrees with the measured radiative rate of LH2 [66].

#### 3.1.5. Fractal Analysis of Exciton Dynamics

Fractal dynamics governs many physiological processes and behavior dynamics with measured signals exhibiting self-similarity and scaling [76,77]. Self-similarity refers to that the fluctuation patterns at faster time scales imitate those at slower time scales. Scaling means that the pattern measurements depend on the resolution or the time scale at which the measurements are conducted [76,77]. Many methods for fractal analysis have been proposed and applied to study the scaling rules in the signals from various processes [78]. A recently developed method by Gao and coworkers, called adaptive fractal analysis (AFA), has been widely adopted to establish the scaling rules of many signals [77,79,80,81,82,83]. Optimally combining local linear or polynomial fitting, the AFA method can obtain a globally smooth trend and mitigate the discontinuities between adjacent data fragments, making it suitable to deal with arbitrarily strong nonlinear trends [77]. The latter is especially pertinent to analyzing simulated signals here, as they all show strong oscillatory trends.

In the AFA algorithm, a global trend, $\nu \left(i\right),i=1,2,\ldots ,N_{f}$, is firstly determined by partitioning original data u(i) into windows of length w=2n+1 and fitting the data in each window with polynomial of order *M* (M=1 in this work). Two neighboring windows overlap with n+1 points. The fit to the overlapping points is achieved by a weighted combination of the fitting polynomials of two adjacent fragments. Then the variance F(w) of the fitting residual u(i)−ν(i) is computed to yield the Hurst parameter *H* with the scaling equation

(19) $$F\left(w\right)={\left[\frac{1}{N_{f}}\sum_{i=1}^{N_{f}}{\left(u(i)-\nu (i)\right)}^{2}\right]}^{1/2}∼w^{H}.$$

In the fractional Brownian motion (fBm) and the fractional Gaussian noise (fGn) processes, the value of *H* falls within the interval of [0,1]. 0<H<1/2, H=1/2 and 1/2<H<1 correspond to the processes with anti-persistent correlation, short-range correlation, and persistent long-range correlation, respectively. Beyond the fBm-fGn framework, a variety of processes have H>1, such as the center of pressure signals, the on-off intermittent and the Lévy walk processes [77,82,83,84]. These are the superdiffusive processes in physics and chemistry.

**Figure 6:**
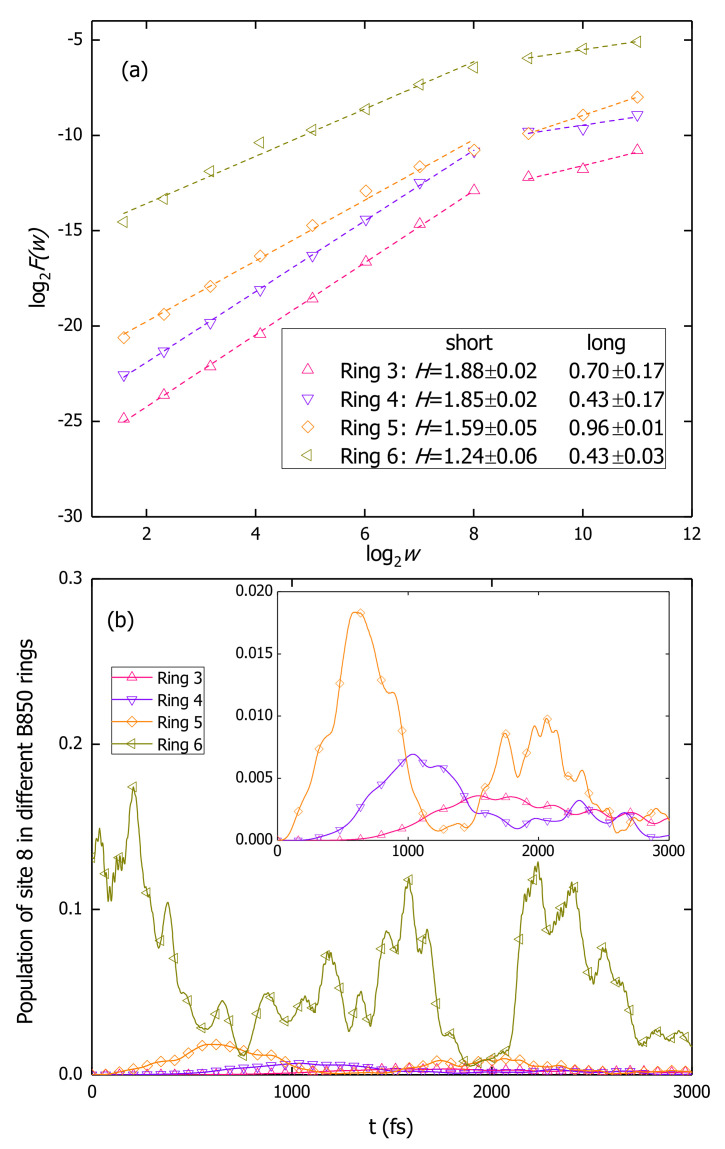
(**a**) Results of the adaptive fractal analysis for the population dynamics of site 8 in different B850 rings. The dashed lines in (**a**) are the linear fitting curves with the slopes (Hurst parameter *H*) shown in the legend. (**b**) Exciton populations on site 8 in four rings (rings 3, 4, 5, and 6). The simulation is carried out for a B850 chain containing 11 rings and initially the central ring (ring 6) is excited to its second lowest exciton state. The insert in (**b**) is the enlarged plot of the population on site 8 in rings 3, 4, and 5.

**Figure 7:**
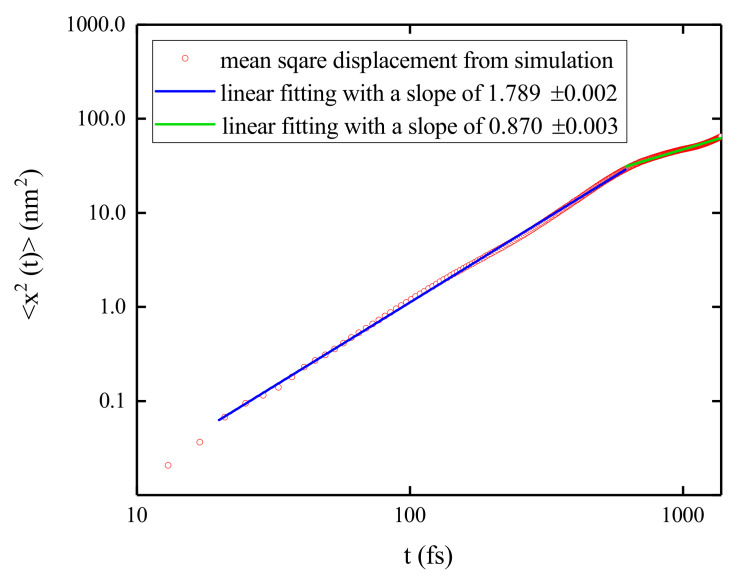
Bilogarithmic plot of the mean square displacement as a function of time for the exciton diffusion in a B850 chain containing 11 rings. The open circles are the results from the simulation which is initiated from the second lowest exciton state of the central ring (ring 6). The red (green) line is the linear fitting curve for the short (long) time regime. The fitting slopes are shown in the legend.

In this work, the AFA method is applied to analyze the population dynamics during the exciton diffusion in B850 nanoarrays and the curves of log${}_{2}$F(w) vs. log${}_{2}$*w* are shown in Figure 6a. A simulation for a B850 chain containing 11 rings is conducted with the second lowest exciton state of the central ring (ring 6) initially populated. Shown in Figure 6b, the populations on site 8 in four rings (rings 3, 4, 5, and 6) are extracted and used as the source data for the AFA. Both high and low frequency oscillations are present in the exciton populations. The fast oscillating patterns resemble those with low oscillation frequencies, indicating the temporal self-similarity of the exciton populations. Fitting the data points with linear functions, one can obtain the Hurst parameters *H* from fitting slopes, which are shown in the legend of Figure 6a. For short times, i.e., $w<2^{8}=256$ fs, the Hurst parameters larger than 1 indicate superdiffusive exciton dynamics in B850 nanoarrays. From the bilogarithmic plot of the MSD versus time shown in Figure 7, a superdiffusion component is found to last for about 600 fs with a γ≈1.789. Persisting for about 250 fs, the scaling regime with H>1 is nearly half of the duration of superdiffusion and this phenomenon is consistent with the fact that the fractal scaling usually only extends to half of the period of the signal [79]. Once the window size *w* exceeds $2^{8}+1=257$ fs, the slopes exhibit obvious decreases compared with those in short times. Determined by the fluctuation amplitudes of the population, F(w) for the site 8 in ring 6 has the largest absolute value among all cases. The Hurst parameter for ring 6 is found to be the smallest due to phonon induced decoherence.

#### 3.1.6. Exciton Diffusion in 2D B850 Nanoarray

**Figure 8:**
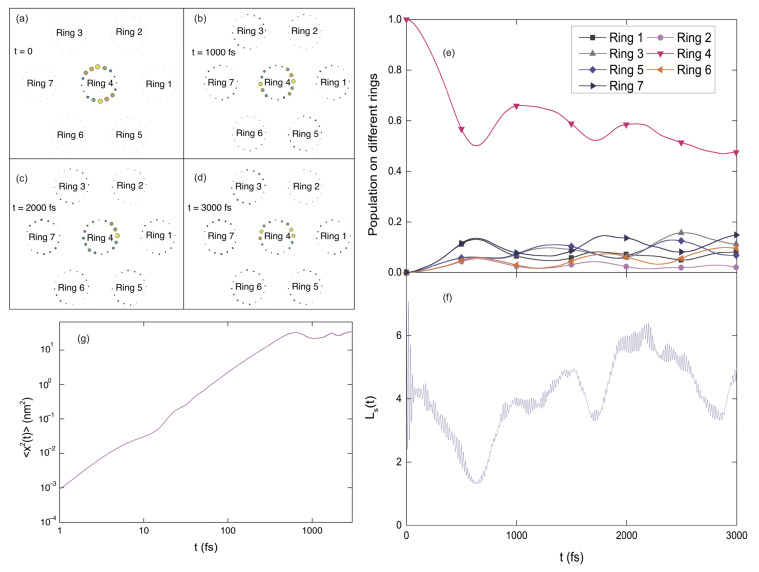
Exciton diffusion in a 2D nanoarray composed of 7 B850 rings. (**a**–**d**) Four snapshots of excitation probability distribution among all the pigments. The radii of the color bubbles represent the excitation probability on individual pigments. (**e**) Population dynamics on all the B850 rings. (**f**) Time evolution of the superradiance enhancement factor $L_{s}$. (**g**) log-log plot of the mean square displacement versus time.

In addition to the B850 chains, the exciton diffusion in 2D nanoarrays of B850 rings is also studied in this section. As shown in Figure 8a, one central ring (ring 4) is surrounded by six rings, and the initial excitation is on the central ring. As time progresses, the excitation energy is gradually transferred to the surrounding rings. Three snapshots of the excitation probability distribution on all the pigments during the dynamics are shown in Figure 8b–d. The time-dependent populations in all B850 rings are depicted in Figure 8e. By comparing the central-ring populations for the 1D (Case II in Figure 4a) and the 2D (Ring 4 in Figure 8e) B850 nanoarrays, more efficient exciton transfer is found in the 2D nanoarray, because of more available energy-transfer channels in the 2D nanoarray than the B850 chain. Similar to the 1D case, extremely efficient energy transfer occurs in the first 600 fs with about half of the excitation energy migrates out from the central ring to the adjacent rings in the 2D nanoarray. During this time interval, the exciton propagates with superdiffusive behavior, which can be seen from Figure 8g where $\langle x^{2}\rangle \propto t^{1.66}$ within 600 fs. Recently, anomalous triplet exciton diffusion for short times is also observed in the single tetracene crystals [85]. Similar to the scenario in the B850 chain, the exciton is delocalized over about 4 pigments in the 2D nanoarray as indicated by the superradiance enhancement factor $L_{s}$ in Figure 8f. This means that the exciton delocalization size is invariant with respect to the system dimension, while the excitation energy transfer is more efficient in the 2D nanoarrays than that in the B850 chains.

### 3.2. Incoherent Exciton Hopping in B850 Nanoarrays

In realistic photosynthetic systems, LHCs are positioned randomly on chromatophore membranes. The electronic coupling between the pigments from different complexes is very weak, and large coherence sizes across different complexes, obtained under ideal circumstances, are rarely achieved. The environmental fluctuations also destroy coherence and induce dissipation. Therefore, it is reasonable to model the B850 complex as one single aggregate by neglecting intra-ring energy transfer, thereby allowing for the application of the Förster theory to excitation energy propagation in large B850 nanoarrays by treating inter-aggregate energy transfer as incoherent exciton hopping. Therefore, the results from the classical hopping method are also presented in this section to provide a rough overview of the exciton diffusion in 1D and 2D systems, as a useful comparison to the fully quantum approach.

As described above, the excitonic superdiffusive behavior only persists for about 500 fs, and the exciton is only transferred over a short distance from the initial ring of excitation within this time interval [see the MSD shown in Figure 4c and Figure 8g]. Therefore, this short-time superdiffusion is ignored in this section, and the classical master equation is applied to simulate exciton diffusion in B850 nanoarrays. In this model, the excitation energy transfer in B850 nanoarrays is determined by a few control parameters, i.e., the decay rate *K*, the inter-ring transfer rate *k*, the distance *a* between adjacent B850 rings, and the parameters of the excitation laser. In this study, a fixed inter-ring distance a=8 nm is chosen and the effects of all the other parameters on the exciton diffusion in B850 nanoarrays will be investigated in this section.

**Figure 9:**
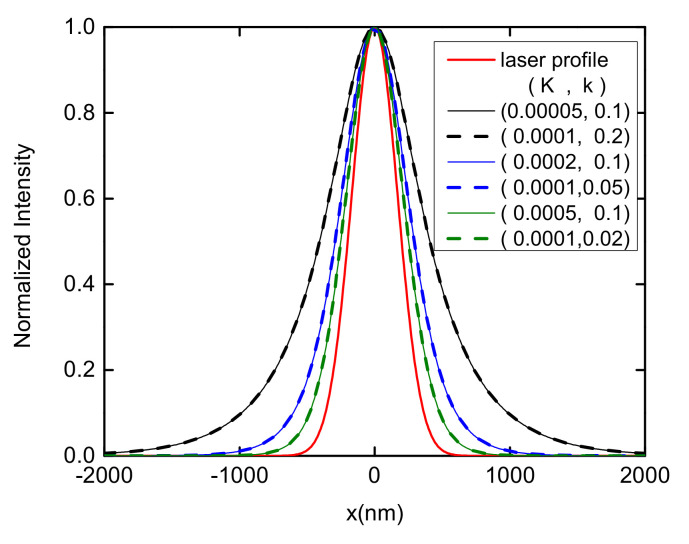
Normalized stationary excitation distribution in a B850 chain with different decay rates *K* and transfer rates *k*. The first parameter in the bracket is the value of the decay rate and the second one is for the transfer rate. Both *K* and *k* are in units of ps${}^{-1}$. The solid red line is the excitation laser profile which is centered at x=0 and has a full width of half maximum (FWHM) of 400 nm. The other three solid lines are the results with the same *k* and dashed lines are those with the same *K*. Each pair of solid and dash lines with the same color have the same K/k ratio.

For native LH2 complexes, the inter-complex excitation energy transfer rate *k* is estimated to 0.1 ps${}^{-1}$[86,87]. The decay rate *K* is chosen as the inverse of the fluorescence life time of the singlet excited state of BChl*a*, $K\approx 10^{-3}$ ps${}^{-1}$[7,66,87,88,89,90]. Firstly, the influences of the decay rate *K* and the transfer rate *k* on the normalized stationary excitation distribution (NSED) in a B850 chain are investigated and the results are shown in Figure 9. A Gaussian laser profile $A_{i}=A_{0}\exp\left[-{\left[\left(i-i_{0}\right)a\right]}^{2}/2\sigma^{2}\right]$ is adopted with a standard deviation (SD) σ=169.86 nm, which leads to a FWHM of 400 nm. The laser is centered at complex $i_{0}$ which locates at x=0, and $A_{0}$ is set to unity throughout this study. The B850 chain contains 4001 rings to mimic a system of an infinite size and to ensure that the stationary distribution is achieved before the exciton reaches the system boundary.

**Figure 10:**
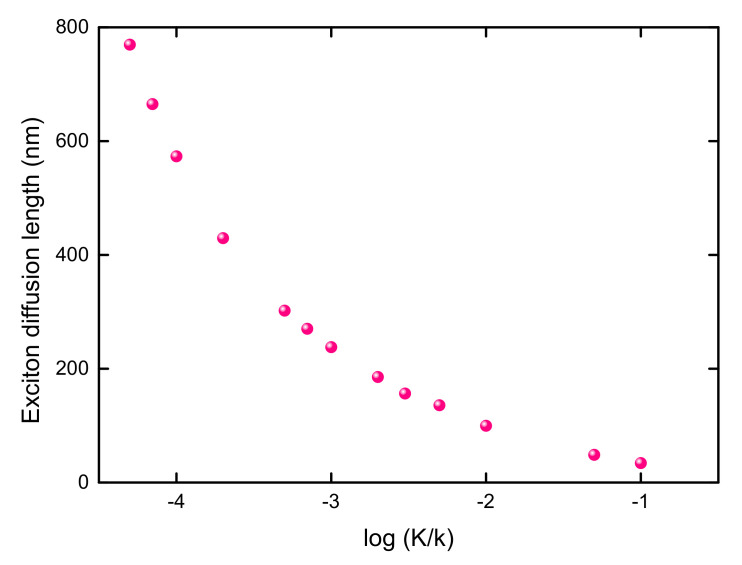
The influence of K/k ratio on the exciton diffusion length in 1D B850 nanoarray. The center of the laser profile is at x=0 and the FWHM of the laser is 400 nm.

As shown in Figure 9, the excitation energy transfer is accelerated (decelerated) by increasing the transfer rate *k* (the decay rate *K*). As expected, the NSED only depends on the ratio of K/k for a fixed laser profile. This phenomenon can be clearly seen from comparing the solid and dashed lines of the same color in Figure 9. With a fixed K/k ratio, various sets of (*K*, *k*) can produce the same NSED. It can be concluded that the energy transfer in the 1D B850 nanoarray is determined by K/k. In order to demonstrate the dependence of the excitation energy transfer on K/k, the exciton diffusion length $L_{d}=\sqrt{\sigma_{s}-\sigma }$ is calculated, where $\sigma_{s}$ (σ) is the SD of the stationary excitation probability distribution (the laser profile) [85,91]. As shown in Figure 10, there is an obvious increase in the exciton diffusion length with a decrease in K/k. As K/k steadily decreases, $L_{d}$ tends to infinity. If *K* is comparable with *k*, the exciton diffusion length is relatively short. This dependence of the energy transfer length on K/k is important to design of artificial photosynthetic devices. Comparing to the exciton diffusion distance in native light-harvesting systems which is less than 50 nm [12], a longer exciton diffusion length of 100 nm is obtained with the parameters of $K=10^{-3}\;{\mathrm{ps}}^{-1}$ and $k=0.1\;{\mathrm{ps}}^{-1}$, as disorder is neglected in the calculations.

In addition to the stationary excitation probability distribution, the time-dependent excitation probability in both 1D and 2D B850 nanoarrays is further investigated in this section. The 1D and 2D systems contain 501 and 2601 B850 rings, respectively. The initial excitation is prepared with a Gaussian laser profile with σ = 33.97 nm and placed at the center of the nanoarrays, i.e., an initial condition of $p_{m}\left(0\right)=c_{1}\exp\left[-d_{m}^{2}/2\sigma^{2}\right]$ ($p_{lm}\left(0\right)=c_{2}\exp\left[-d_{lm}^{2}/2\sigma^{2}\right]$) for the 1D (2D) sample. $c_{1}$ ($c_{2}$) is the normalization constant for the initial excitation probability and $d_{m}$ ($d_{lm}$) is the distance between the central ring and ring *m* [(l,m)] in the 1D (2D) nanoarray. In the calculations, the decay rate *K* and the transfer rate *k* are 0.001 ps${}^{-1}$ and 0.1 ps${}^{-1}$, respectively.

**Figure 11:**
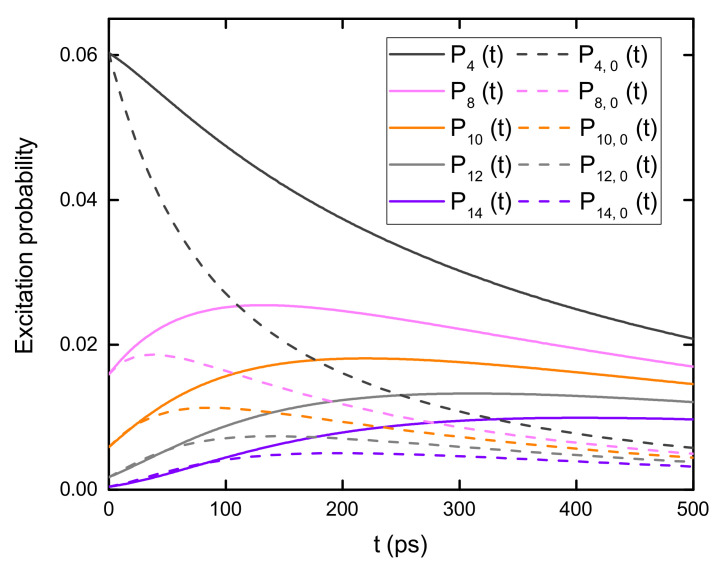
Comparison of the excitation probability dynamics for B850 rings in 1D model (solid lines) and 2D nanoarray (dashed lines). The 1D and 2D models contain 501 and 2601 B850 rings, respectively. The initial excitation is at the center of the nanoarrays. Prepared initial conditions are $p_{m}\left(0\right)=c_{1}\exp\left[-d_{m}^{2}/2\sigma^{2}\right]$ for 1D sample and $p_{lm}\left(0\right)=c_{2}\exp\left[-d_{lm}^{2}/2\sigma^{2}\right]$ for 2D sample with σ=33.97 nm. Constant $c_{1}$ ($c_{2}$) is used to normalize the initial excitation probability and $d_{m}$ ($d_{lm}$) is the distance between ring *m* [(l,m)] and the central ring in the 1D (2D) system. The decay rate $K=0.001\;ps^{-1}$ and the transfer rate $k=0.1\;ps^{-1}$.

To investigate the influence of nanoarray structures on the excitation energy transfer in B850 nanoarrays, one can compare the time evolution of the excitation probability of B850 rings in 1D and 2D nanoarrays with the results shown in Figure 11. The excitation probabilities of five rings in the 1D nanoarray (solid lines) are compared with those in the 2D nanoarray (dashed lines). The distance between ring *m* and ring 0 in the B850 chain is equivalent to that between ring (m,0) and ring (0,0) in the 2D nanoarray. For ring *m* in 1D array and ring (m,0) in 2D array, the results are displayed with the same color. The excitation probability of ring (m,0) in the 2D model is scaled to produce an initial excitation probability identical to that of ring *m* in the 1D system.

As shown is Figure 11, the excitation probability decreases monotonically with time if the ring is close to the center of the initial excitation profile, i.e., ring 0 [ring (0,0)] in the 1D (2D) nanoarray. For a ring located far away from the laser profile center, the probability first increases for short times and then decreases steadily. The short-time increase of the excitation probability is mainly due to the incoming excitation energy transferred from the ones next to the center of the laser profile. The probability decrease at long times stems from energy migration to other distant rings. Direct evidence of the energy transfer is the delayed maximum excitation probability for rings located further from the center of the initial excitation profile. Due to the irreversible decay of the excitation energy, the maximum probability decreases with the increased distance between the ring and the center of the laser profile. The excitation probabilities for the rings in the 2D system change more rapidly than that for the 1D sample. In addition, the maximum probabilities in the 2D case is lower than that in the 1D case, a result stemmed from the fact that there are more available channels for energy transfer in the 2D nanoarray.

## 4. Conclusions

Combining the Dirac–Frenkel time-dependent variation with the Davydov trial states and implementing a numerically efficient algorithm on the GPU architecture, we present a fully quantum mechanical description of the exciton–phonon dynamics in mesoscale photosynthetic systems. Excellent performance has been achieved in modeling the exciton diffusion in huge B850 nanoarrays containing more than 1600 pigments. With the advanced GPU algorithm, the computational time is substantially reduced relative to the conventional computation bared on CPUs. It is found that initially populating the second lowest exciton state (optically bright) of the B850 ring leads to more efficient inter-ring energy transfer than other initial excitation patterns. This phenomenon is due to a large transition dipole moment of the optically bright state of the B850 ring and is consistent with the previous finding that an exciton state with a larger transition dipole moment can give rise to more efficient excitation energy transfer [75]. The superradiance enhancement factor is calculated revealing an exciton delocalization over 2-3 BChls, in agreement with the measurements of fluorescence quantum yield and lifetime [66].

Both high and low frequency oscillations are present in the dynamics of exciton populations, exhibiting characteristic fractal dynamics which has been widely observed in many physiological processes and behavior dynamics. With the exciton dynamics obtained by the aforementioned fully quantum mechanical method, a fractal analysis is performed for the first time to unveil the self-similarity between the high and low frequency oscillations in the exciton dynamics. Such temporal self-similarity is found in the exciton populations, as the fast oscillating patterns resemble those with low oscillation frequencies. Through the fractal analysis, a superdiffusive component is identified to persist for about 500 fs in the exciton dynamics, in agreement with an analysis of the exciton MSD.

Due to weak inter-ring coupling and decoherence, the exciton diffusion in B850 nanoarrays can also be described with the classical master equation by treating each B850 ring as an aggregate. We obtain analytical expressions of the time-dependent excitation probability in both 1D and 2D nanoarrays. The ratio between the decay rate *K* and the transfer rate *k* is found to determine the stationary excitation probability distribution. From both the coherent and incoherent descriptions, the exciton transfer in 2D B850 nanoarrays is more efficient than that in B850 chains, thanks to multiple channels involved in the energy transfer process.

Taking advantage of the GPU architecture, we have successfully applied the time dependent variational method with the Davydov *Ansatz* to present a fully quantum mechanical description of exciton–phonon dynamics in mescoscale photosynthetic systems consisting of more than 1600 molecules. Our work sheds light on excitation energy transfer in realistic photosynthetic systems composed of LH2, LH1-RC complexes, such as the recently reported 100-million-atom scale model of an entire chromatophore vesicle [13]. Investigations in this direction are in progress.

Recently, 2D layered transition metal dichalcogenide (TMD) nanosheets have attracted a great amount of attention due to their diversity, accessibility, and versatile and tuneable properties [92]. Mechanisms underlying excitonic diffusion and relaxation in TMD monolayers are fundamentally and technological intriguing problems. The methodology we have developed here in this work can be applied to tackle various issues related to TMD excitonic diffusion, such as phonon scattering and quantum confinement. Work in this direction is also currently in progress.

## Data Availability

The data presented in this study are available on request from the corresponding author.

## Appendix A. Exciton Hamiltonian of B850 Nanoarrays

Each B850 ring is composed of 8 BChl*a* dimers. Two pigments within in each dimer are labeled as BChl*a* α and BChl*a* β, respectively. The site energy of BChl*a* α (BChl*a* β) is $\epsilon_{\alpha }=$ 12,050 cm${}^{-1}$ ($\epsilon_{\beta }=$ 11,970 cm${}^{-1}$) [33]. The off-diagonal elements in *J* are the excitonic coupling strengths between different pigments. The intra-dimer coupling strength is 205 cm${}^{-1}$ and the coupling strength between BChl*a* α and BChl*a* β in adjacent dimer is 144 cm${}^{-1}$. The excitonic coupling strength between the BChl*a* α (BChl*a* β) molecules in two neighboring dimers is −52 cm${}^{-1}$ (-31 cm${}^{-1}$) [33]. The coupling strengths between the other pairs of pigments are estimated with the transition charges from the electrostatic potentials method [71,93,94]. Shown in Table A1 is the exciton Hamiltonian of a B850 ring used in our simulations.

**Table A1:** Exciton Hamiltonian of a B850 ring (in unit of cm${}^{-1}$).

Site	1	2	3	4	5	6	7	8	9	10	11	12	13	14	15	16
1	$\epsilon_{\alpha }$	205	−52	14.4	−7	2.9	−2.2	1.2	−1.4	1.2	−2.2	2.9	−7	14.4	−52	144
2		$\epsilon_{\beta }$	144	−31	14.4	−4	2.9	−0.9	1.2	0.4	1.2	−0.9	2.9	−4	14.4	−31
3			$\epsilon_{\alpha }$	205	−52	14.4	−7	2.9	−2.2	1.2	−1.4	1.2	−2.2	2.9	−7	14.4
4				$\epsilon_{\beta }$	144	−31	14.4	−4	2.9	−0.9	1.2	0.4	1.2	−0.9	2.9	−4
5					$\epsilon_{\alpha }$	205	−52	14.4	−7	2.9	−2.2	1.2	−1.4	1.2	−2.2	2.9
6						$\epsilon_{\beta }$	144	−31	14.4	−4	2.9	−0.9	1.2	0.4	1.2	−0.9
7							$\epsilon_{\alpha }$	205	−52	14.4	−7	2.9	−2.2	1.2	−1.4	1.2
8								$\epsilon_{\beta }$	144	−31	14.4	−4	2.9	−0.9	1.2	0.4
9									$\epsilon_{\alpha }$	205	−52	14.4	−7	2.9	−2.2	1.2
10										$\epsilon_{\beta }$	144	−31	14.4	−4	2.9	-0.9
11											$\epsilon_{\alpha }$	205	−52	14.4	−7	2.9
12												$\epsilon_{\beta }$	144	−31	14.4	−4
13													$\epsilon_{\alpha }$	205	−52	14.4
14														$\epsilon_{\beta }$	144	−31
15															$\epsilon_{\alpha }$	205
16																$\epsilon_{\beta }$

## Appendix B. Derivation of Exciton-Phonon Dynamics in B850 Nanoarrays

In the framework of the Dirac–Frenkel time-dependent variational principle [49], the Lagrangian *L* is written as

(A1) $$L=\langle \Psi_{D_{1}}\left(t\right)|\frac{i\hbar }{2}\left(\frac{\vec{\partial }}{\partial t}-\frac{\overset{\leftarrow }{\partial }}{\partial t}\right)-\hat{H}|\Psi_{D_{1}}\left(t\right)\rangle .$$

Applying the Lagrangian *L* to the Euler-Lagrange equations

(A2) $$\begin{array}{ccc} \frac{d}{dt}\left(\frac{\partial L}{\partial \dot{\alpha_{m}^{r*}}}\right)-\frac{\partial L}{\partial \alpha_{m}^{r*}} & = & 0 \end{array}$$

(A3) $$\begin{array}{ccc} \frac{d}{dt}\left(\frac{\partial L}{\partial \dot{\lambda_{mq}^{r*}}}\right)-\frac{\partial L}{\partial \lambda_{mq}^{r*}} & = & 0, \end{array}$$

we can obtain the equations of motions for the time-dependent variational parameters $\alpha_{m}^{r}\left(t\right)$ and $\lambda_{mq}^{r}\left(t\right)$:

(A4) $${\dot{\alpha }}_{m}^{r}\left(t\right)=i\left[T_{m}^{r}\left(t\right)+\alpha_{m}^{r}\left(t\right)R_{m}^{r}\left(t\right)\right],$$

(A5) $${\dot{\lambda }}_{mq}^{r}\left(t\right)=i\left[\frac{\Omega_{mq}^{r}\left(t\right)}{\alpha_{m}^{r}\left(t\right)}+\frac{g_{q}^{r}}{\sqrt{N}}\omega_{q}^{r}e^{-iqm}-\omega_{q}^{r}\lambda_{mq}^{r}\left(t\right)\right],$$

with

(A6) $$T_{m}^{r}\left(t\right)=-\sum_{r^{\prime }}\sum_{n}J_{mn}^{rr^{\prime }}\alpha_{n}^{r^{\prime }}\left(t\right)S_{mn}^{rr^{\prime }}\left(t\right),$$

(A7) Rmr(t)=Re∑q−Ωmqr(t)αmr(t)+gqrNωqre−iqmλmqr*(t),

and

(A8) $$\begin{array}{ccc} \Omega_{mq}^{r}\left(t\right) & = & -\sum_{r^{\prime }}\sum_{n}J_{mn}^{rr^{\prime }}\alpha_{n}^{r^{\prime }}\left(t\right)S_{mn}^{rr^{\prime }}\left(t\right)\left[\lambda_{nq}^{r^{\prime }}\left(t\right)\delta_{rr^{\prime }}-\lambda_{mq}^{r}\left(t\right)\right]. \end{array}$$
